# The Role of Methylation Analysis in Distinguishing Cellular Myxoma from Low-Grade Myxofibrosarcoma

**DOI:** 10.3390/ijms25105105

**Published:** 2024-05-08

**Authors:** Hanna Henzinger, Iva Brčić, Jasminka Igrec, Theresa Marie Godschachner, Susanne Scheipl, Joanna Szkandera, Philipp Jurmeister, Bernadette Liegl-Atzwanger

**Affiliations:** 1 Diagnostic and Research Institute of Pathology, Medical University of Graz, 8010 Graz, Austria; hanna.henzinger@medunigraz.at (H.H.); theresa.godschachner@medunigraz.at (T.M.G.); bernadette.liegl-atzwanger@medunigraz.at (B.L.-A.); 2 Division of General Radiology, Department of Radiology, Medical University of Graz, 8010 Graz, Austria; jasminka.igrec@medunigraz.at; 3 Department of Orthopedics and Trauma, Medical University of Graz, 8010 Graz, Austria; susanne.scheipl@medunigraz.at; 4 Division of Clinical Oncology, Department of Medicine, Medical University of Graz, 8010 Graz, Austria; joanna.szkandera@medunigraz.at; 5 Institute of Pathology, Ludwig Maximilians University Hospital Munich, 80336 Munich, Germany; 6 German Cancer Consortium (DKTK), Partner Site Munich, 80336 Munich, Germany; 7 German Cancer Research Center (DKFZ), 69120 Heidelberg, Germany

**Keywords:** cellular myxoma, intramuscular myxoma, myxofibrosarcoma, methylation analysis, *GNAS* mutation

## Abstract

Cellular myxoma is a benign soft tissue tumor frequently associated with *GNAS* mutation that may morphologically resemble low-grade myxofibrosarcoma. This study aimed to identify the undescribed methylation profile of cellular myxoma and compare it to myxofibrosarcoma. We performed molecular analysis on twenty cellular myxomas and nine myxofibrosarcomas and analyzed the results using the methylation-based DKFZ sarcoma classifier. A total of 90% of the cellular myxomas had *GNAS* mutations (four loci had not been previously described). Copy number variations were found in all myxofibrosarcomas but in none of the cellular myxomas. In the classifier, none of the cellular myxomas reached the 0.9 threshold. Unsupervised t-SNE analysis demonstrated that cellular myxomas form their own clusters, distinct from myxofibrosarcomas. Our study shows the diagnostic potential and the limitations of molecular analysis in cases where morphology and immunohistochemistry are not sufficient to distinguish cellular myxoma from myxofibrosarcoma, particularly regarding *GNAS* wild-type tumors. The DKFZ sarcoma classifier only provided a valid prediction for one myxofibrosarcoma case; this limitation could be improved by training the tool with a more considerable number of cases. Additionally, the classifier should be introduced to a broader spectrum of mesenchymal neoplasms, including benign tumors like cellular myxoma, whose distinct methylation pattern we demonstrated.

## 1. Introduction

Intramuscular/cellular myxoma is a benign mesenchymal tumor typically located in skeletal muscles of the proximal extremities, most commonly the thigh. This tumor frequently affects adults aged 40 to 70 and is more commonly seen in females. Usually, the tumor shows slow growth, is circumscribed but unencapsulated and has an excellent prognosis, with rare recurrences after surgical excision.

Histologically, intramuscular myxoma consists of bland eosinophilic spindle- to stellate-shaped cells embedded in an abundant myxoid, “frothy” stroma, which may contain microcystic spaces, vacuolated macrophages and few capillary-sized blood vessels. Nuclear atypia, pleomorphism, or necrosis are not seen, and mitoses are scarce. While classic intramuscular myxoma is a hypocellular lesion, the cellular subtype of intramuscular myxoma shows hypercellular areas occupying 10–90% of the tumor [1]. This feature is accompanied by a more prominent capillary network with occasional thick-walled vessels as well as a focally more collagenous background. For resection specimens, histologic diagnosis is usually not a major challenge. On a molecular level, myxomas show recurrent *GNAS* mutations in up to 90% of cases. In contrast, low-grade MFS is a hypocellular multinodular tumor with incomplete fibrous septa, abundant myxoid stroma, non-cohesive spindle and stellated cells with eosinophilic cytoplasm, atypical enlarged nuclei and typical curvilineal thin-walled blood vessels with perivascular condensation of tumor cells [1,2].

Nowadays, pathologists are expected to diagnose soft tissue tumors using limited biopsy material. Especially in this setting, the differential diagnosis between intramuscular/cellular myxoma and a deep-seated low-grade myxofibrosarcoma (MFS) is essential, as MFS, in contrast to intramuscular/cellular myxoma, has a high local recurrence rate in 30–40% of cases and therefore requires an oncologic treatment strategy [1,3].

This study focuses on the value of analyzing the molecular alterations in twenty cases of cellular myxoma using different molecular techniques. We aimed to evaluate the feasibility of performing methylation analysis on cellular myxomas and to identify DNA methylation patterns specific to this entity. In addition, we compared the DNA methylation profiles of cellular myxomas with nine cases of low-grade MFS and to already published data.

## 2. Results

### 2.1. Clinical Data

The clinical data of the cellular myxoma and MFS cases were very similar to the reported epidemiological data and are summarized in Table 1. There was a female predominance (60% and 75%, respectively), and the mean patient age was 58 years for cellular myxoma (ranging from 39 to 90 years) and 67 years for MFS (ranging from 44 to 88 years). The mean size of the cellular myxomas was 5.4 cm, ranging from 1.8 to 12 cm, while the mean size of MFS was 6.3 cm, ranging from 1.3 to 10 cm. The cellular myxomas were localized predominantly in the lower extremity (15/20 cases, 75%), eleven (55%) in the thigh and four (20%) in the gluteal region. Four tumors (20%) were located in the upper extremity and shoulder, and one (5%) in the thoracic wall. MFSs were also most commonly located in the lower extremity (5/8, 62.5%), with one tumor (12.5%) in the thigh and four in the calf (50%). Two MFSs (25%) occurred in the soft tissues of the upper back (scapular region) and one (12.5%) in the right flank. (Tables S1 and S2) One of the analyzed cellular myxomas (case 16), with classic morphology, was a recurrence of a cellular myxoma resected 9.5 years prior. A total of 18/20 (90%) cellular myxomas were intramuscular, one (5%) was subcutaneous and one (5%) was deeply seated in the thorax wall. In contrast, 5/8 (62.5%) MFSs were subcutaneous lesions and 3/8 (37.5%) were deeper subfascial masses.

### 2.2. Radiological, Histological and Immunohistochemical Findings

**Figure 1:**
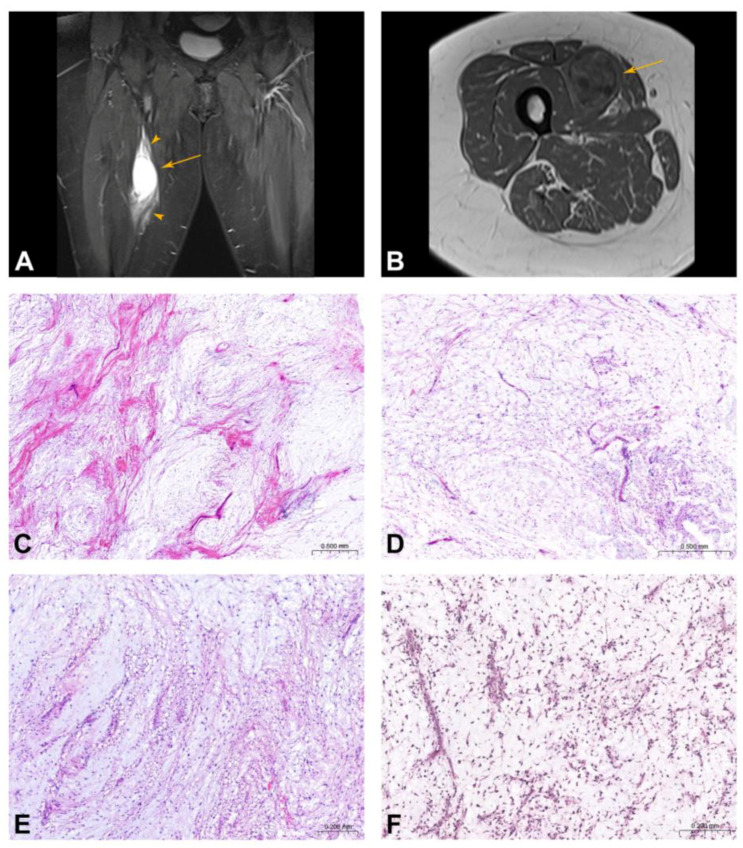
Radiological and histological features. (**A**) Myxoma of the right thigh in a 66-year-old female patient. On the T1-weighted coronal MRI sequence, a hyperintense oval lesion (arrow) with perilesional oedema along the muscle fascia (arrowhead). (**B**) Heterogenous slow lesion enhancement on T1-weighted sequence after gadolinium application corresponding to myxoid tissue. (**C**,**D**) Cellular myxoma comprises bland spindle and stellate cells embedded in an abundant myxoid and collagenous stroma. In between, scattered capillary-sized blood vessels are found. (**E**) Focally, vacuolated macrophages are seen. (**F**) Low-grade myxofibrosarcoma is composed of abundant myxoid stroma containing scattered atypical hyperchromatic cells and curvilinear blood vessels.

MRI was available for 15 (75%) cases of cellular myxoma and all (100%) MFS cases. Cellular myxomas were hypointense in T1-weighted images and hyperintense in T2-weighted images with minimal to distinct, sometimes patchy or peripheral contrast enhancement (Figure 1A,B). Perifocal oedema was described in 2/15 (13.3%) cases. MFSs were multinodular, with areas of low to intermediate signal intensity on both T1- and T2-weighted images with heterogeneous and avid enhancement after contrast administration and in five cases, the tail sign was present.

**Figure 2:**
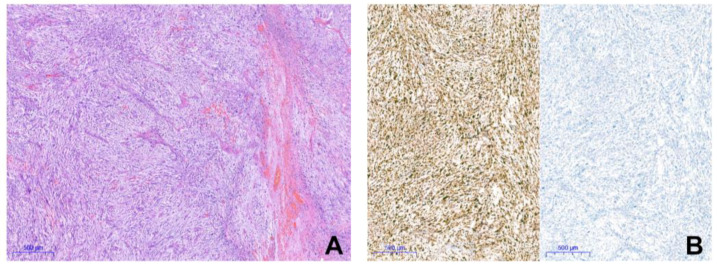
Myxofibrosarcoma case with MDM2 amplification. (**A**) Tumor is composed of atypical spindle cells with prominent vasculature in a myxoid to collagenous background. (**B**) Immunohistochemical findings: MDM2 (**left**) is positive, CDK4 (**right**) is negative.

All cellular myxomas were composed of bland eosinophilic spindle to stellate-shaped cells and scattered capillary-sized blood vessels embedded in an abundant “frothy” myxoid and collagenous stroma (Figure 1C,D). Focally, microcystic spaces and vacuolated macrophages (Figure 1E) were found. Areas of increased cellularity in 10–90% of the sampled tumor areas, more prominent capillary vessels, occasional thick-walled vessels as well as a focal collagenous background were seen. All MFSs classified as G2 on the resection specimen had a low-grade component, composed of abundant myxoid stroma containing non-cohesive scattered atypical hyperchromatic cells and curvilinear blood vessels with atypical cells centered around them (Figure 1F). Immunohistochemically, all cellular myxomas (20/20, 100%) and all MFSs (9/9, 100%) showed multifocal positivity for CD34. All tumors were negative for S100, SOX10 and MUC4. In two MFS cases (8a and 8b) a focal to multifocal expression of MDM2 was seen, while CDK4 staining was negative (Figure 2).

### 2.3. Mutational Analysis of Cellular Myxomas

A total of 18/20 (90%) cases of cellular myxoma had *GNAS* mutations located in exon 8:9 (45%) on the locus c.G602A:p.R201H, 5 (25%) on c.601 (4/5 c.C601T:p.R201C and 1/5 c.C601A:p.R201S), 2 (10%) on c.2530 (c.C2530T:p.R844C and c.C2530A:p.R844S) and 1 (5%) each on c.A610G:p.T204A and c.G2531A:p.R844H, respectively (Table 2). The average variant frequency (VAF) of the myxomas with *GNAS* mutation was 10.67% (ranging from 2.35% to 27.91%). We also detected additional mutations in *TSC2* (2/20; exon33:c.G3889A:p.A1297T, VAF 44.8%; exon13:c.T1301C:p.I434T, VAF 50.7%), *PTCH1* (1/20; exon14:c.C2173T:p.P725S, VAF 57.7%) and *TP53* (1/20; exon4:c.G329A:p.R110H, VAF 56%) (Supplementary Figure S1). However, according to publicly available databases (Varsome, ClinVar), all additional mutations (including *TP53*) were classified as benign/likely benign and therefore seem to have no biological relevance. The two cases lacking *GNAS* mutation were reevaluated, and despite a tumor content adequate for analysis, no mutations were found.

In total (including consult cases of one of the authors BLA), 47 cases of intramuscular myxoma were diagnosed at the D&R Institute of Pathology between 2017 and 2023. In 32 cases (68.1%) the diagnosis of a “cellular myxoma” was given. A total of 37 cases (78.7%) tested positively for *GNAS* mutation, 8 cases (17%) were negative and 2 (4.3%) samples did not contain enough DNA for NGS.

### 2.4. Copy Number Variation (CNV) Analysis

No copy number alterations were found in any of the eleven myxoma samples containing enough DNA. In contrast, CNV analysis of all MFSs detected gains and losses on different chromosomes, as listed in Table 3. In detail, *MTCP1* amplification and *RB1*, *CYSLTR2* and *MLLT3* deletions were each found in two (22.2%) of the nine samples, and focal deletion of *CDKN2A* occurred in three cases (33.3%). The two tumor samples (primary tumor and recurrence) of one patient (cases 8a and 8b) both demonstrated *PTPRB* and *MDM2* amplification (these cases were reevaluated to rule out liposarcoma; see Section 3). Additional amplification of *WT1*, *ERC1*, *ASIC2* and *MTCP1* and deletion of *CDKN2A* and *MLLT3* were found in recurring MFS (case 8b) but not in the original tumor (case 8a). The CNV plots of the nine MFS cases and one myxoma case are depicted in Supplementary Figure S2.

### 2.5. Matching the Cases with the DKFZ Sarcoma Classifier

All MFS cases, and 11/20 (55%) cellular myxoma cases contained enough DNA for DNA methylation analysis. Only one (5%) of these samples received a calibrated score greater than 0.9, defined as the threshold for a valid result of methylation-based tumor classifying tools [4]. The successfully classified case was an MFS sample assigned to the “undifferentiated sarcoma” methylation class. Considering classifications with a calibrated score between 0.3 and 0.9, seven (77.8%) of the MFS samples were classified as “undifferentiated sarcoma” and two (22.2%; one tumor was a local recurrence in the same patient) were classified as “epithelioid sarcoma” (immunohistochemistry performed in these two cases demonstrated intact INI1 (SMARCB1) staining). None of the eleven cellular myxoma cases received a classifier score > 0.9. The most commonly predicted methylation classes with a classifier score between 0.3 and 0.9 were “well-/dedifferentiated liposarcoma” (5/11; 45%), followed by “myositis proliferans” (3/11; 27%), “undifferentiated sarcoma” (2/11; 18%) and “alveolar soft part sarcoma” (1/11; 9%) (Table 4).

**Figure 3:**
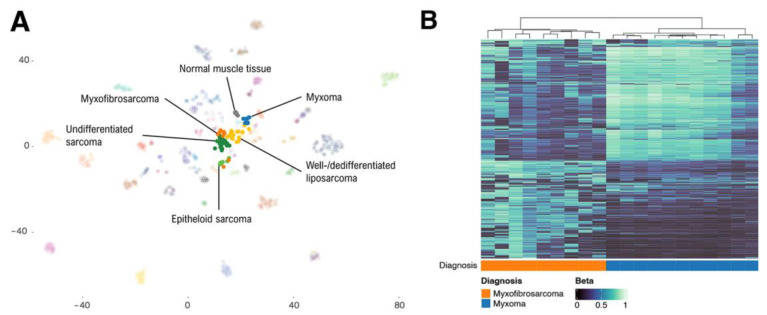
Unsupervised DNA methylation analysis. (**A**) Joint t-distributed stochastic neighbor embedding of cases from the reference cohort used to train the Heidelberg sarcoma classifier and our cellular myxoma and myxofibrosarcoma cases. Cases with MDM2 amplification clustered with epithelioid sarcomas. (**B**) Unsupervised clustering shows a clear separation of cellular myxomas and myxofibrosarcomas.

Unsupervised t-SNE analysis of our cases combined with the reference cohort of the sarcoma classifier is shown in Figure 3A. All myxomas, including the two *GNAS* wild-type cases, aggregated in a separate class close to liposarcoma. The observations for the MFS cases were in line with the classifier results as seven aggregated near samples from the “undifferentiated sarcoma” class and two near “epithelioid sarcoma” cases. Hierarchical clustering of our myxoma and MFS cases showed a clear separation between the tumor entities (Figure 3B).

### 2.6. Fish

FISH was performed on two MFS samples (8a and 8b) to confirm that these cases are not dedifferentiated liposarcomas (as suggested by methylation analysis). Consistent with the findings of CNV analysis and immunohistochemistry, the cases showed *MDM2* amplification in FISH, while *CDK4* amplification was absent.

## 3. Discussion

The study analyzed the value of molecular analysis to differentiate between cellular myxoma and low-grade MFS on limited biopsy material. As we all know, limited biopsy material in combination with intratumoral morphologic heterogeneity commonly seen in soft tissue tumors can complicate the correct diagnosis. In addition, low-grade myxofibrosarcoma (MFS) and cellular myxoma cannot be separated via immunohistochemistry, as common CD34 expression is seen in both tumor entities.

Intramuscular/cellular myxoma is a tumor that occurs sporadically as well as in patients with Mazabraud syndrome, presenting as one or more intramuscular or cellular myxomas in combination with fibrous dysplasia [1,3]. The tumors show recurrent *GNAS* mutations in up to 90% of cases [1,2]. So far, mutations in *GNAS* exon 8 on positions 601 and 602 have been described in intramuscular myxoma [5]. In contrast, MFSs are among the tumors with complex karyotypes and often display inter- and intratumoral genetic heterogeneity [6,7]. Alongside frequent mutation of *TP53* and cell cycle checkpoint genes, copy number variations (CNV) are typically observed in these tumors and have been described in *ATRX*, *CCND1*, *CCNE1*, *CDH1*, *CDK6*, *CDKN2A*, *CDKN2B*, *EGFR*, *EPHA3*, *EPHB1*, *FGFR1*, *FOXA1*, *GNAS*, *HDLBP*, *JAK1*, *JUN*, *KRAS*, *MDM2*, *NF1*, *NKX2–1*, *NTRK1*, *PTEN*, *RB1*, *RET*, *SYK*, *TP53* and *WNT11* [6,7,8]. The reported molecular alterations in MFSs are of limited use in the daily routine workup.

Although limited biopsy material and a low tumor cell content are making molecular testing difficult, we attempted to evaluate a targeted approach using small NGS mutational panel as well as methylation analysis (including CNV analysis) in this context [1,3]. Using the former, we found *GNAS* mutations in 18 of our 20 cases of cellular myxomas (90%), in keeping with the previous findings [2]. All *GNAS* mutations were located in exon 8, 14 in previously described loci p.R201C, p.R201S and p.R201H [5]. In addition, we demonstrate four novel, not previously described *GNAS* mutations in loci p.T204A, p.R844C, p.R844S and p.R844H. The relatively small VAF of *GNAS* mutations in our tumor samples (on average 10.7%, ranging from 2.35% to 27.91%) match our observation that the tumors contain mostly fibroblasts (supportive cells) and endothelial cells, with only a small percentage of tumor cells.

In everyday work, targeted mutational analysis works well on core needle biopsies. In our study, almost half of tested cellular myxomas did not contain enough DNA for subsequent methylation analysis. This suggests the limited use of this method in a routine diagnostic setting. Of note, putative mutations called at ≤5% VAF are frequently due to sequencing errors; therefore, reporting these mutations bears the risk of false positive results. In our study, 3/18 (16.67%) cases had VAF < 5%.

As expected, we found no CNV in any of the myxoma cases, confirming the benign nature of these tumors. This finding suggests that all myxomas showed a simple karyotype; however, these should be taken with caution, as only a limited NGS panel was used in our study. In contrast, in all MFS samples amplifications and deletions were detected. The most frequent CNV in our cohort was the focal deletion of *CDKN2A*. In summary, 6/9 (66.7%) of the MFSs had alterations in genes related to regulating the G1/S cell cycle checkpoint (*CDKN2A*, *CCND1* and RB1). *RB1* deletions occurred together with *CYSLTR2* deletions in 2/9 (22.2%) cases; the latter has yet to be described in MFSs. In addition, we found further not previously described alterations in 29 other genes, namely, *AFF3*, *ARID2*, *ASIC2*, *ATP1A1*, *BCLAF1*, *BIRC3*, *BTK*, *CDH11*, *COX6C*, *CYLD*, *ECT2L*, *ERC1*, *LCP1*, *MACC1*, *MLLT3*, *MTCP1*, *MYB*, *NFATC1*, *NOTCH2*, *PABPC1*, *PAX7*, *POU2AF1*, *PTBRB*, *RGS22*, *SGK1*, *SORBS2*, *STAG1*, *TNFAIP3* and *WT1*.

The two MFS samples obtained from one patient had *PTPRB* and *MDM2* amplification. We confirmed *MDM2* amplification using FISH; however, these tumors harbored no CDK4 co-amplification, a finding already described in MFSs by Ogura et al. [7]. Furthermore, the recurring case (case 8b) had gained additional copy number alterations in addition to the sample from the primary resection.

In recent years, there has been an increased effort to classify mesenchymal tumors using DNA methylation analysis, and a machine learning-based sarcoma classifier has been established by the German Cancer Research Center (DKFZ) in Heidelberg in 2021 [9]. However, while this classifier tool is very useful for sarcoma research, it still needs to be integrated into routine pathology practice since the algorithm only includes 45 distinct mesenchymal tumor entities, leaving many others, like intramuscular/cellular myxoma, unrepresented [8,10]. As machine learning classifiers tend to assign cases from unknown classes to the most similar class, the authors of the sarcoma classifier implemented calibrated probability scores to reduce the risk of this type of misclassification. As expected, none of our myxoma cases exceeded the cut off for a clear classification (0.9), but most had intermediate scores between 0.3 and 0.9; this was most frequently the case for well-/dedifferentiated liposarcoma. Scores in this range can still be considered valid after careful examination, as they can also occur in cases with low tumor cell content or bad overall sample quality. Of note, our myxoma cases showed none of the morphologic features of atypical lipomatous tumors/well-differentiated liposarcomas. (Supplementary Figure S1) In addition, 90% of cases harbored a *GNAS* mutation.

In the Heidelberg sarcoma classifier, MFSs are part of the methylation class “undifferentiated sarcoma” (USARC), a group that also comprised undifferentiated pleomorphic sarcoma and pleomorphic liposarcoma [9]. Of our nine histologically validated MFSs, seven samples were assigned to the “undifferentiated sarcoma” class, although only one case had a classifier score > 0.9. The two samples that occurred in the same patient (cases 8a and 8b) were classified as epithelioid sarcomas. Upon reevaluation, we confirmed that both tumors had a pure spindle cell morphology and intact nuclear INI-1 expression and were negative for keratin, EMA, and ERG. The tumor showed focal expression of CD34. Morphology and IHC profile did not match with the diagnosis of epithelioid sarcoma [11]. Nevertheless, mutations in regulators of the epigenome have also been described in MFSs, for example *ATRX*, a member of the SWI/SNF family of chromatin remodeling proteins, which is a potential explanation for clustering near epithelioid sarcoma.

Unsupervised t-SNE analysis using cases from the reference cohort of the sarcoma classifier showed that our myxoma cases aggregated in a separate group that was close but markedly distinct from the well-/dedifferentiated liposarcoma class. Furthermore, myxomas were distinct from MFS cases, and unsupervised clustering separated these entities. This shows that cellular myxomas and MFSs have distinct DNA methylation profiles that could be used to differentiate these entities in challenging diagnostic cases. This might be particularly helpful for *GNAS* wild-type tumors.

The average calibrated score of the MFS samples was higher than that of the myxomas but still too low for a valid prediction in 88.9% of the cases, which points to the limitations of the classifier in its current version. The main goal should be to reinforce the classifier by more cases and to expand the classifier by additional soft tissue tumor entities listed in the WHO classification to enhance the current version into a valuable diagnostic tool. This could lower the discrepancies between histological diagnosis and DNA methylation-based classification observed in 17% of cases in a validation study [12]. The rate reported in the respective study was lower (12%) if only entities represented in the classifier were considered. This study also showed that the algorithm’s accuracy varies between different sarcoma types, e.g., of the MFSs evaluated, only 42.9% were correctly predicted to the USARC class “undifferentiated sarcoma” [12]. Of note, as the MFSs cluster in the group of undifferentiated sarcomas, the classifier cannot distinguish UPSs from MFSs.

As RNA-based NGS techniques and targeted mutational panels have an impact in soft tissue tumor pathology and work well on limited biopsy material, the methylation classifier could have a valuable diagnostic impact, especially for tumor entities lacking recurrent genetic changes.

Our study was limited by the small number of evaluated cases. In addition, almost half of the myxoma samples did not contain enough DNA for DNA methylation analysis, further narrowing the study collective. However, based on the hypocellularity of intramuscular myxomas and low-grade MFSs, a limited DNA content is expected.

In conclusion, our results support the previous finding that intramuscular cellular myxoma harbors *GNAS* mutation in up to 90% of cases, distinguishing it from MFSs [2]. Furthermore, in our myxoma cohort we found novel *GNAS* mutation loci on exon 8. In challenging cases, *GNAS* mutation analysis is of diagnostic value in daily routine to differentiate intramuscular/cellular myxoma from malignant mimickers. We also showed that cellular myxomas harbor no copy number alterations, a finding that could help in complex cases, especially biopsy material. In addition, we found 30 new amplifications and deletions in our MFS cohort underlining the complex genetic changes in this tumor and highlighting that morphology in combination with immunohistochemistry remains the golden standard in diagnosing this tumor entity. DNA methylation analysis showed that cellular myxomas have a distinct DNA methylation profile that allows separation from MFSs; however, methylation profiling is not yet reliably applicable in routine diagnostic setting.

## 4. Materials and Methods

### 4.1. Histologic Characteristics

In total, twenty cases of cellular myxoma were prospectively tested for *GNAS* mutations between 2017 and 2022. Nineteen myxomas were resected, while for one case (case 5), only biopsy material was available. To evaluate the molecular profile of a potential mimicker, nine MFS resection specimens diagnosed between 2016 and 2021 were retrieved from the surgical pathology files of our institution. Of the MFSs we analyzed, one case was graded as G1 and eight cases were graded as G2 in the pathology report, according to FNCLCC grading system. The grade 1 MFS was a hypocellular multinodular tumor with abundant myxoid stroma, non-cohesive spindle and stellated cells with eosinophilic cytoplasm and atypical enlarged nuclei and typical curvilinear thin-walled blood vessels with perivascular condensation of tumor cells. The mitotic activity was below 2 per mm^2^. Grade 2 MFSs showed, in addition to clear cut areas of G1 morphology, hypercellular areas with increased mitotic activity. The mitotic activity ranged between 6 and 9 per mm^2^. However, the tumors lacked solid tumor areas and necrosis. From the G2 tumors, only low-grade areas were selected for molecular analysis and manually microdissected.

The histology of all cases was reviewed by two soft tissue pathologists (IB and BLA) prior to inclusion, and tumor areas were marked for further analysis. In the extensive sampled material, no components of atypical lipomatous tumor (ALT)/well-differentiated liposarcoma were detected. The inclusion criteria for MFS G2 cases were a sufficient number of low-grade areas in non-prior treated surgical specimens to ensure the extraction of an adequate amount of DNA from tumor tissue for mutational analysis, CNV and DNA methylation analysis.

All cases were selected from the surgical pathology files of the D&R Institute of Pathology, Medical University of Graz. The clinical data (patient age and gender, tumor size and location, radiology reports) were retrieved from the medical files. One MFS sample (case 8b) was a local recurrence of case 8a. Molecular and methylation analyses were performed on both cases separately, but the patient characteristics and tumor localization were only included once in the clinical data statistic. At the time of diagnosis, two patients diagnosed with cellular myxoma (case 6, case 17) had two separate tumors in the same anatomic area. In these cases, only one sample was taken for molecular and methylation analysis, given the identical morphology.

Additionally, the pathology reports of all cases of intramuscular myxoma (including the cellular variant) diagnosed at our institution between 2017 and 2023 were retrieved from our hospital database to determine the frequency of *GNAS* mutations in intramuscular/cellular myxoma in a single institution.

Institutional ethical approval was obtained from the Institution Review Board (EK Number 34–115 ex 21/22). Samples used for this project have been provided by Biobank Graz, Austria. Informed consent was obtained.

### 4.2. Immunohistochemistry

For immunohistochemical analysis, we used formalin-fixed, paraffin-embedded (FFPE) material. Immunohistochemistry (IHC) was performed on 4 µm thick whole tissue sections. We used the Benchmark Ultra platform (Ventana Medical Systems, Tucson, AZ, USA) with iVIEW DAB Detection Kit (Ventana Medical Systems) using assays for the evaluation of two antibodies: anti-human mouse monoclonal antibody MUC4 (1:30, clone 8G7, Sigma Aldrich, St. Louis, MO, USA) and SOX10 (RTU, clone SP267, Sigma Aldrich). S100 (RTU, Dako/Agilent, Glostrup, Denmark) and CD34 (RTU, clone QBEnd10, Dako/Agilent) immunohistochemistry was performed using the Dako Omnis platform. MDM2 (1:30, clone 0846, Calbiochem, San Diego, CA, USA) and CDK4 (1:100, clone sc-23896, Santa Cruz Biotechnology, Dallas, TX, USA) staining was performed on three MFS cases (cases 6, 8a and b). Specific cytoplasmic and/or membranous and a nuclear, moderate-to-strong staining pattern (depending on the antibody) in >1% of tumor cells were considered positive.

### 4.3. Molecular Analysis

Genomic DNA of twenty cellular myxoma cases was extracted from FFPE material (6–8 unstained, 10 µm thick sections). H&E-stained sections of FFPE blocks were examined, and in cases of resection specimens, areas of higher tumor cell content were marked and used for analysis. DNA isolation was performed using the Maxwell RSC DNA FFPE kit (Promega, Madison, WI, USA) according to the manufacturer’s instructions. DNA was quantified by PicoGreen fluorescence. 20 ng DNA were used to perform next-generation sequencing (NGS)-based anchored multiplex PCR technique (Ion Torrent S5, MUG Soft tissue Panel searching for mutations in the *BRAF*, *CTMMB1*, *GNAS*, *PDGRFA*, *GNAQ*, *MED12*, *MYOD1*, *PIK3CA*, *KIT*, *TP53*, *FH*, *PTCH1*, *NF1*, *NF2*, *RB1*, *PRKAR1A*, *TSC1* and *TSC2* genes). Mutations were annotated following the recommendations of the Human Genome Variation Society (HGVS, http://varnomen.hgvs.org/, accessed on 8 January 2024), Varsome (https://varsome.com/, accessed on 8 January 2024) and ClinVar (https://www.ncbi.nlm.nih.gov/clinvar/, accessed on 8 January 2024).

### 4.4. DNA Methylation Analysis

DNA methylation analysis was performed using the Illumina Infinium MethylationEPIC BeadChip technology (Illumina, Inc., San Diego, CA, USA) using at least 500 ng DNA. DNA from our FFPE samples was prepared using the Illumina Infinium HD FFPE DNA Restore Kit, and bisulfite conversion was carried out using the EpiTect Bisulfite Kit (Qiagen, Hilden, Germany). After amplification, fragmentation and hybridization of the DNA with the Illumina Infinium MethylationEPIC BeadChip, an Illumina NextSeq 550 device was used for scanning of the arrays. The results were analyzed with the DKFZ sarcoma classifier (www.molecularsarcomapathology.org, accessed on 8 January 2024). Copy number profiles were derived from raw DNA methylation data using a modified version of the conumee package that automatically detects focal copy number alterations as previously described [13,14].

Due to the overall limited tumor cell content, 9/20 cases (45%) of intramuscular myxoma did not contain enough DNA for further analysis; therefore, we only reviewed the clinical data and reported the detected *GNAS* point mutations of these nine cases. The DNA methylation patterns of the eleven remaining myxoma cases were evaluated and compared to those of the nine MFS samples.

Raw DNA methylation data for the reference cohort of the DKFZ sarcoma classifier were obtained from the Gene Expression Omnibus database. Statistical analysis was performed using RStudio Pro 2022.12.0+353.pro20 based on R version 4.1.2. Joint preprocessing of all samples was performed using a modified version of the minfi package. The pfilter and dasen function from the wateRmelon package were used to filter bad-quality samples and probes and for normalization and preprocessing. Cross-reactive probes and probes associated with SNPs and sex chromosomes were excluded, and the 20,000 most variants CpGs were selected for dimensionality reduction using the t-distributed stochastic neighbor embedding (t-SNE) method.

### 4.5. Fish

For FISH analysis, the ZytoLight^®^ SPEC MDM2/CEN 12 Dual Color Probe and the ZytoLight^®^ SPEC CDK4/CEN 12 Dual Color Probe were used according to the manufacturer’s protocol. Cases were scored by counting a minimum of 40 nuclei per case under oil immersion at 100 magnification with a DAPI/green/red triple band pass filter. A ratio > 2.0 was considered amplified for the *MDM2* and/or *CDK4* gene.

## Figures and Tables

**Table 1:** Summary and comparison of clinical data.

		Cellular Myxoma (n = 20)	Myxofibrosarcoma (n = 8)
Sex	Male	8 (40%)	2 (25%)
	Female	12 (60%)	6 (75%)
Age at diagnosis	Mean (±SD)	57.95 (13.65)	67.00 (13.12)
	95% CI	51.97–63.93	57.91–76.09
	Range	39–90	44–88
Dimension (cm)	Mean (±SD)	5.40 (2.58)	6.3 (3.28)
	95% CI	4.32–6.48	4.19–8.47
	Range	1.8–12	1.3–10
Multiple tumors (2)	No. of Patients	2 (10%)	0 (0%)
Location	Left	6 (30%)	5 (62.5%)
	Right	13 (65%)	3 (37.5%)
	No data	1 (5%)	0 (0%)
	**Upper extremity**	4 (20%)	2 (25%)
	Scapula	1 (5%)	2 (25%)
	Upper arm	2 (10%)	0 (0%)
	Lower arm	1 (5%)	0 (0%)
	**Lower extremity**	15 (75%)	5 (62.5%)
	Glutes	4 (20%)	0 (0%)
	Thigh	11 (55%)	1 (12.5%)
	Calf	0 (0%)	4 (50%)
	**Other**	1 (5%)	1 (12.5%)
	Thoracic wall	1 (5%)	0 (0%)
	Flank	0 (0%)	1 (12.5%)

**Table 2:** List of point mutations in cellular myxoma.

GNAS	18	90%
**exon8:c.601**	**5**	**25%**
exon8:c.C601T:p.R201C	4	20%
exon8:c.C601A:p.R201S	1	5%
**exon8:c.602**	**9**	**45%**
exon8:c.G602A:p.R201H	9	45%
**exon8:c.610**	**1**	**5%**
exon8:c.A610G:p.T204A	1	5%
**exon8:c.2530**	**2**	**10%**
exon8:c.C2530T:p.R844C	1	5%
exon8:c.C2530A:p.R844S	1	5%
**exon8:c.2531**	**1**	**5%**
exon8:c.G2531A:p.R844H	1	5%
***TSC2***	**2**	**10%**
**exon13:c.1301**	**1**	**5%**
exon13:c.T1301C:p.I434T	1	5%
**exon33:c.3889**	**1**	**5%**
exon33:c.G3889A:p.A1297T	1	5%
***PTCH1***	**1**	**5%**
**exon14:c.2173**	**1**	**5%**
exon14:c.C2173T:p.P725S	1	5%
***TP53***	**1**	**5%**
**exon4:c.329**	**1**	**5%**
exon4:c.G329A:p.R110H	1	5%

**Table 3:** Focal copy number variations in myxofibrosarcoma.

Case No.	Amplifications	Deletions
1	*CCND1*, *MTCP1*	*SORBS2*
2	*SGK1*, *BCLAF1*, *MYB*, *TNFAIP3*, *ECT2L*, *MACC1*, *PABPC1*, *RGS22*, *COX6C*	*CYSLTR2*, *RB1*, *BTK*
3	*ARID2*	*RB1*, *CYSLTR2*
4	*BIRC3*, *POU2AF1*, *CDH11*	*PAX7*, *CYLD*, *NFATC1*, *CDKN2A*, *MLLT3*
5	*AFF3*, *STAG1*	*CDKN2A*
6	*-*	*LCP1*
7	*JUN*, *ATP1A1*, *NOTCH2*	*-*
8a	*PTPRB*, *MDM2*	*-*
8b	*WT1*, *ERC1*, *PTPRB*, *MDM2*, *ASIC2*, *MTCP1*	*CDKN2A*, *MLLT3*

**Table 4:** Results of methylation analysis.

Case No.	Diagnosis	Methylation Class	Calibrated Score	Interpretation
1	Myxofibrosarcoma	Undifferentiated sarcoma	0.34121	no match
2	Myxofibrosarcoma	Undifferentiated sarcoma	0.84525	no match
3	Myxofibrosarcoma	Undifferentiated sarcoma	0.75552	no match
4	Myxofibrosarcoma	Undifferentiated sarcoma	0.20334	no match
5	Myxofibrosarcoma	Undifferentiated sarcoma	0.99557	match
6	Myxofibrosarcoma	Undifferentiated sarcoma	0.31455	no match
7	Myxofibrosarcoma	Undifferentiated sarcoma	0.18347	no match
8a	Myxofibrosarcoma	Epithelioid sarcoma	0.37310	no match
8b	Myxofibrosarcoma	Epithelioid sarcoma	0.84463	no match
2	Myxoma	Well-/dedifferentiated liposarcoma	0.60467	no match
3	Myxoma	Well-/dedifferentiated liposarcoma	0.39028	no match
6	Myxoma	Undifferentiated sarcoma	0.16493	no match
8	Myxoma	Well-/dedifferentiated liposarcoma	0.71233	no match
11	Myxoma	Myositis proliferans	0.39191	no match
13	Myxoma	Undifferentiated sarcoma	0.12083	no match
14	Myxoma	Myositis proliferans	0.41064	no match
17	Myxoma	Alveolar soft part sarcoma	0.32563	no match
18	Myxoma	Well-/dedifferentiated liposarcoma	0.60728	no match
19	Myxoma	Well-/dedifferentiated liposarcoma	0.73169	no match
20	Myxoma	Myositis proliferans	0.44161	no match

## Data Availability

All data generated or analyzed during this study are included in this published article and its Supplementary Materials files.

## Supplementary Materials

The supporting information can be downloaded at: https://www.mdpi.com/article/10.3390/ijms25105105/s1.
