# Supplementary material for: The Role of Methylation Analysis in Distinguishing Cellular Myxoma from Low-Grade Myxofibrosarcoma
Source: Int J Mol Sci. 2024 May 8;25(10):5105. doi: 10.3390/ijms25105105 (PMC11121712; doi:10.3390/ijms25105105)
Supplement: Supplementary file 1 [file ijms-25-05105-s001.zip › ijms-2961287-supplementary.pdf]

International Journal of
*Molecular Sciences*

MDPI

# The Role of Methylation Analysis in Distinguishing Cellular Myxoma from Low-Grade Myxofibrosarcoma—Supplementary Data

**Table S1.** Clinical data and mutations of cellular myxoma cases.

| Case No. | Sex | Age at diagnosis | No. of tumors | Max. expansion (cm) | Location | *GNAS* (VAF) | Other mutations (VAF) | CNV |
|---|---|---|---|---|---|---|---|---|
| 1 | F | 80 | 1 | 6 | Thigh l | exon8:c.G602A:p.R201H (9%) | | none |
| 2 | M | 40 | 1 | 5.5 | Upper arm l | exon8:c.G602A:p.R201H (13.5%) | | none |
| 3 | M | 71 | 1 | 3.8 | Scapula r | | | none |
| 4 | M | 53 | 1 | 3.7 | Glutes l | exon8:c.C601T:p.R201C (12%) | | none |
| 5 | F | 90 | 1 | 8.2 | Glutes l | exon8:c.G602A:p.R201H (13.6%) | | none |
| 6 | F | 47 | 2 | 8<br>1.8 | Glutes l | exon8:c.G602A:p.R201H (4%) | | none |
| 7 | F | 64 | 1 | 2.7 | Thigh r | exon8:c.C601T:p.R201C (8.7%) | *TSC2* exon33:c.G3889A:p.A1297T (44.8%) | none |
| 8 | M | 42 | 1 | 5 | Thigh r | exon8:c.A610G:p.T204A (10.5%) | | none |
| 9 | F | 64 | 1 | 7.4 | Thigh r | exon8:c.G2531A:p.R844H (23.6%) | | none |
| 10 | M | 40 | 1 | 10 | Thigh l | exon8:c.G602A:p.R201H (3.3%) | | none |
| 11 | F | 66 | 1 | 3.5 | Thoracic wall | | | none |
| 12 | F | 51 | 1 | 3.8 | Upper arm r | exon8:c.C2530T:p.R844C (9%) | *TSC2* exon13:c.T1301C:p.I434T (50.7%)<br>*PTCH1* exon14:c.C2173T:p.P725S (57.7%) | none |
| 13 | F | 62 | 1 | 11 | Thigh r | exon8:c.C2530A:p.R844S (11.8%) | *TP53* exon4:c.G329A:p.R110H (56%) | none |
| 14 | F | 60 | 1 | 4.3 | Thigh r | exon8:c.G602A:p.R201H (26.1%) | | none |
| 15 | F | 68 | 1 | 3.4 | Lower arm r | exon8:c.C601T:p.R201C (11.4%) | | none |
| 16 | F | 50 | 1 | 3.5 | Thigh r | exon8:c.G602A:p.R201H (7.7%) | | none |
| 17 | F | 39 | 2 | 12<br>9 | Thigh r | exon8:c.G602A:p.R201H (5.8%) | | none |
| 18 | M | 51 | 1 | 5 | Thigh r | exon8:c.C601T:p.R201C (9.9%) | | none |
| 19 | M | 64 | 1 | 4.3 | Glutes r | exon8:c.C601A:p.R201S (3.5%) | | none |
| 20 | M | 57 | 1 | 5.1 | Thigh r | exon8:c.G602A:p.R201H (9.2%) | | none |

F - female; l - left; M - male; r – right; max – maximum; VAF – variant average frequency; CNV – copy number variations.

**Table S2.** Clinical data of myxofibrosarcoma cases.

| Case No. | Sex | Age at diagnosis | No. of tumors | Max. expansion (cm) | Location |
|---|---|---|---|---|---|
| 1 | F | 80 | 1 | 5.6 | Scapula l |
| 2 | F | 62 | 1 | 3.7 | Flank r |
| 3 | M | 54 | 1 | 13 | Thigh l |
| 4 | F | 72 | 1 | 7.2 | Calf l |
| 5 | M | 88 | 1 | 8 | Calf l |
| 6 | F | 65 | 1 | 6.7 | Scapula r |
| 7 | F | 44 | 1 | 10 | Calf r |
| 8a | F | 71 | 1 | 5 | Calf l |
| 8b | | | 1 (recurrence of case 8a) | 1.3 | |

F - female; l - left; M - male; r – right; max – maximum.

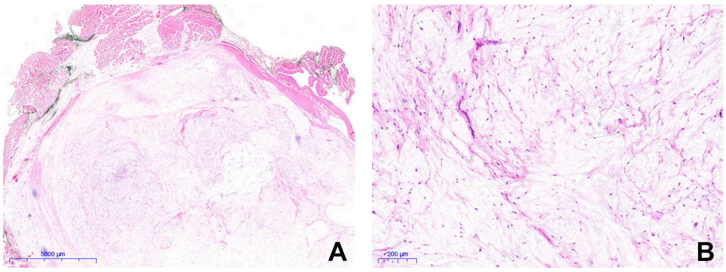


**Figure S1.** Cellular myxoma case with benign *TP53* mutation. (A) Circumscribed tumor mass without infiltration of the adjoining skeletal muscle. (B) Bland spindle cells embedded in a myxoid stroma without necrosis or mitotic activity.

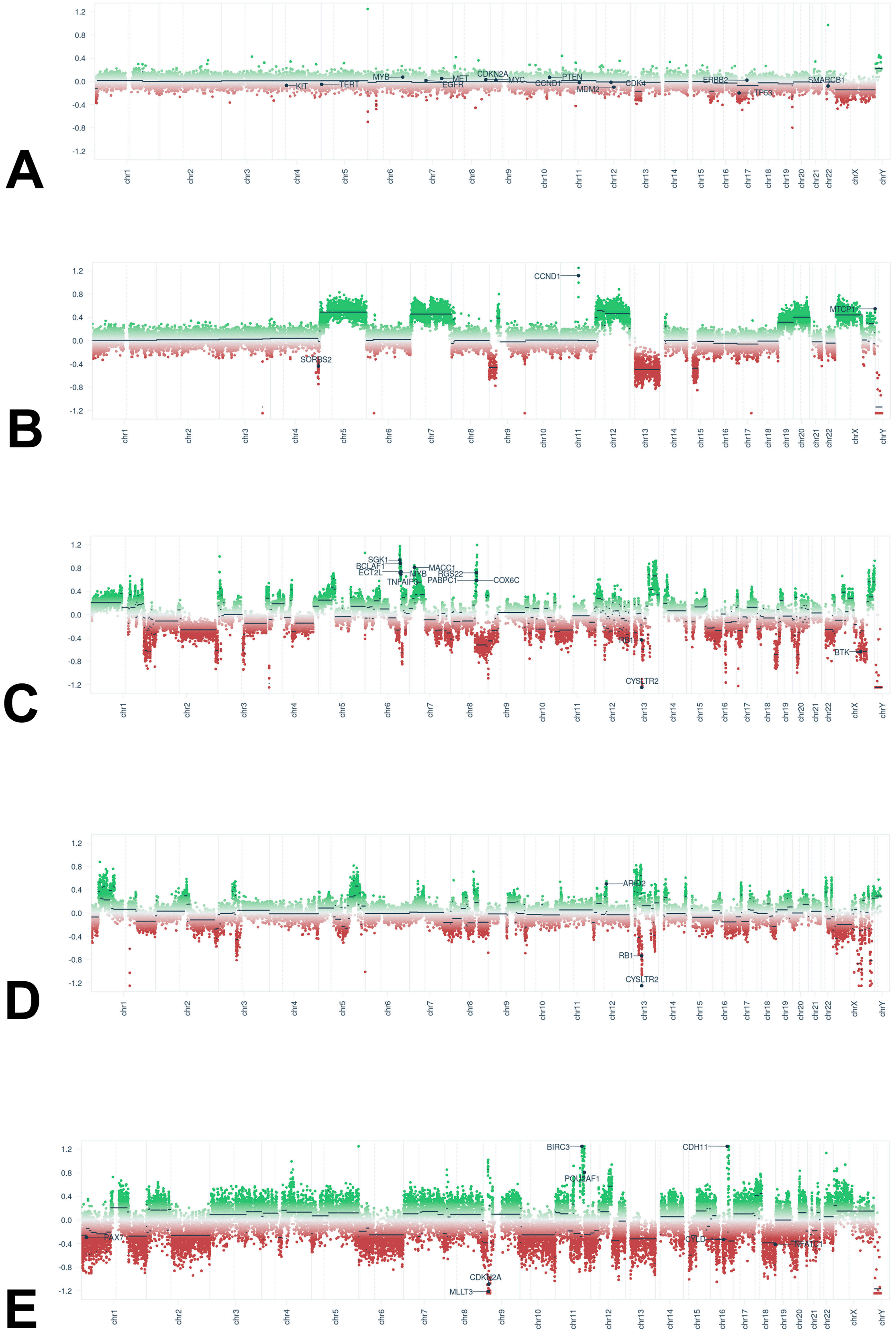
A
B
C
D
E
1.2
0.8
0.4
0.0
-0.4
-0.8
-1.2
chr1
chr2
chr3
chr4
chr5
chr6
chr7
chr8
chr9
chr10
chr11
chr12
chr13
chr14
chr15
chr16
chr17
chr18
chr19
chr20
chr21
chr22
chrX
chrY
KIT
TERT
MYB
MET
EGFR
CDKN2A
MYC
CCND1
PTEN
MDM2
CDK4
ERBB2
TP53
SMARCB1
CCND1
SORBS2
MTCP1
SGK1
BCLAF1
ECT2L
MYB
MACC1
TNFAIP3
RGS22
PABPC1
COX6C
BTK
CYSLTR2
ARID2
RB1
CYSLTR2
BIRC3
POU2AF1
CDH11
PAX7
CYLD
CDKN2A
MLLT3

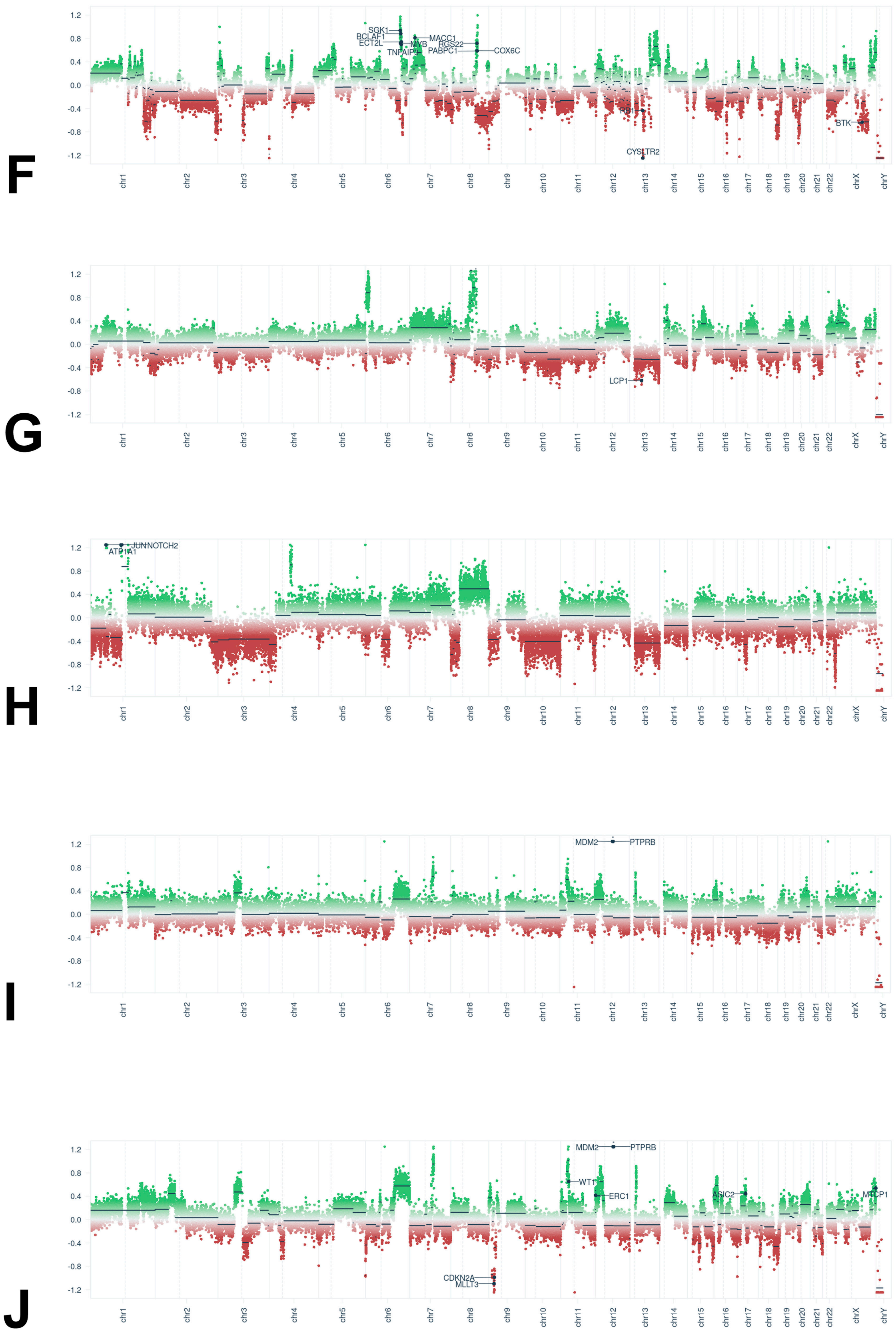


**Figure S2.** CNV plots of (A) one myxoma case and (B-J) all myxofibrosarcoma cases.
